# The phytochemistry and pharmacological activities of *Clinacanthus nutans* (Burm. f.) Lindau: current evidence, mechanistic insights, and future therapeutic perspectives

**DOI:** 10.3389/fphar.2026.1900216

**Published:** 2026-08-25

**Authors:** Wenxuan Si, Xinyao Sun, Yu Han, Yu Zhang, Weiyaun Li, Qi Wei, Qianqian Liu, Muhammad Zahoor Khan, Xia Zhao, Wenqiang Liu

**Affiliations:** 1 College of Agriculture and Biology, Liaocheng University, Liaocheng, China; 2 Liaocheng Academy of Agricultural Sciences, Liaocheng, Shandong, China

**Keywords:** *Clinacanthus nutans*, ethnomedicine, flavonoids, pharmacological activities, phytochemistry, triterpenoids

## Abstract

*Clinacanthus nutans*: a member of the Acanthaceae family native to southern China and Southeast Asia, is a traditional medicinal plant widely used for the treatment of inflammation, viral infections, liver diseases, and metabolic disorders. Despite its long ethnomedicinal history, systematic scientific evaluation remains limited. Recent studies have identified more than 130 active metabolites, including triterpenoids, flavonoids, alkaloids, and sulfur-containing glycosides, which exhibit multiple pharmacological effects such as antioxidant, anti-inflammatory, antiviral, antitumor, hepatoprotective, and immunomodulatory activities. The underlying mechanisms involve multiple targets and signaling pathways, including NF-κB, Nrf2/ARE, AMPK, and apoptosis-related cascades, and clinical applications have included the management of hepatitis, skin infections, rheumatism, and leptospirosis. Safety assessments indicate low acute toxicity, although concerns remain regarding subchronic toxicity and the lack of robust clinical trials. This review systematically examines the botanical characteristics, major chemical metabolites, and pharmacological activities of *C. nutans*, with particular emphasis on recent research progress, mechanistic insights, and the clinical applications of its active metabolites in multifactorial disease interventions. In conclusion, *C. nutans* demonstrates significant medicinal potential, and future research priorities should include bioactivity-guided isolation of novel metabolites, validated *in vivo* mechanistic studies, pharmacokinetic and metabolomic profiling, and the implementation of standardized Phase I/II clinical trials and veterinary evaluations to support its further development and application.

## 1 Introduction

**FIGURE 1:**
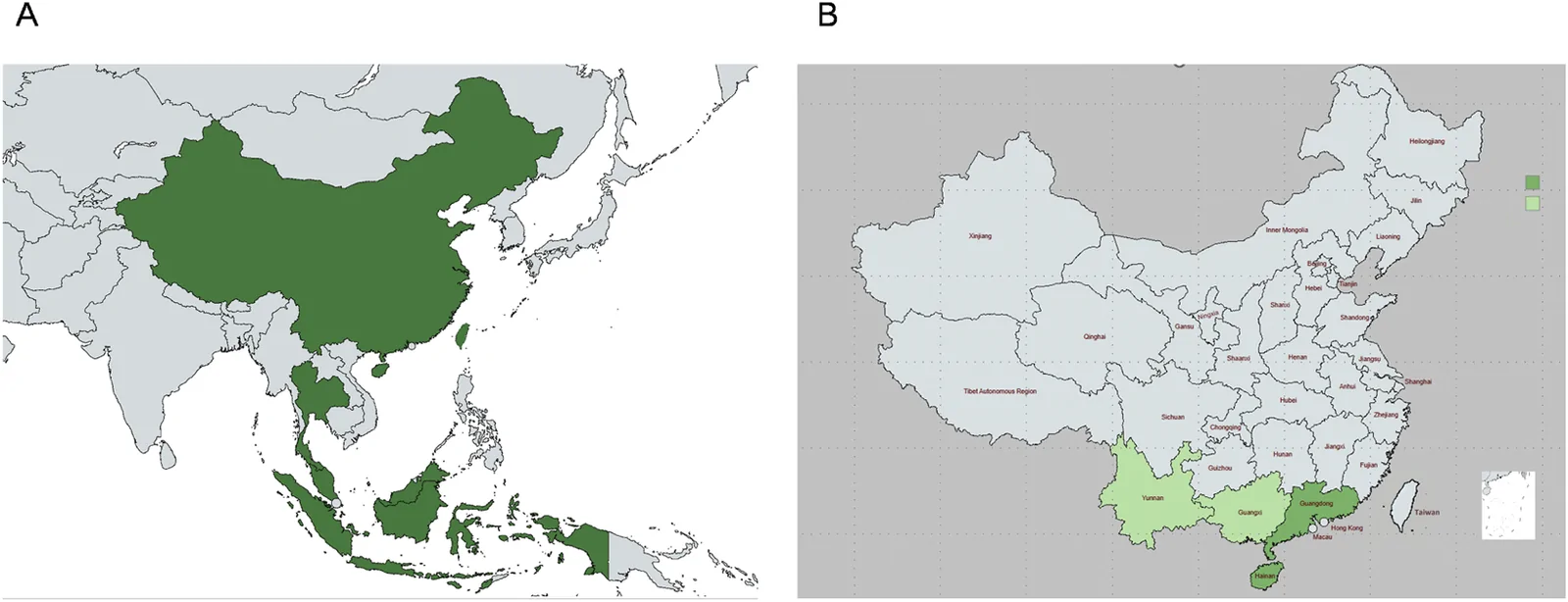
Geographic distribution of *Clinacanthus nutans* and its major ethnomedicinal applications. **(A)** Global distribution map indicating the primary range of C. nutans across China, Malaysia, Indonesia, and Thailand, with inset annotation of key traditional applications by country. **(B)** Provincial distribution within China, highlighting Guangdong, Guangxi, Hainan, and Yunnan as principal growing regions. Colored icons indicate dominant therapeutic applications by region.

*Clinacanthus nutans (Burm. f.) Lindau,* also known as Youduncao, E’zuihua, and Shabashecao—and colloquially as “Sabah snake grass” in Malaysia—belongs to the genus *Clinacanthus* in the family Acanthaceae. It is a perennial medicinal plant widely distributed in southern China (Taiwan, Hainan, Guangdong, Guangxi, Fujian) and Southeast Asia (Malaysia, Thailand, Indonesia) (Figure 1) (Chiangchin et al., 2023).

Traditionally, the leaves are the most commonly used part, with reported functions such as anti-inflammatory and antioxidant effects, reduction of edema, improvement of blood circulation, analgesia, regulation of menstruation, and resolution of blood stasis. In Southeast Asia, *C. nutans* has been employed for skin ailments, snake bites, metabolic disorders, and cancers, reflecting its broad ethnomedicinal relevance (Lin et al., 2023). In Malaysian folk medicine, it is particularly regarded as an anticancer plant and often used as adjuvant therapy (Lin et al., 2023).

**FIGURE 2:**
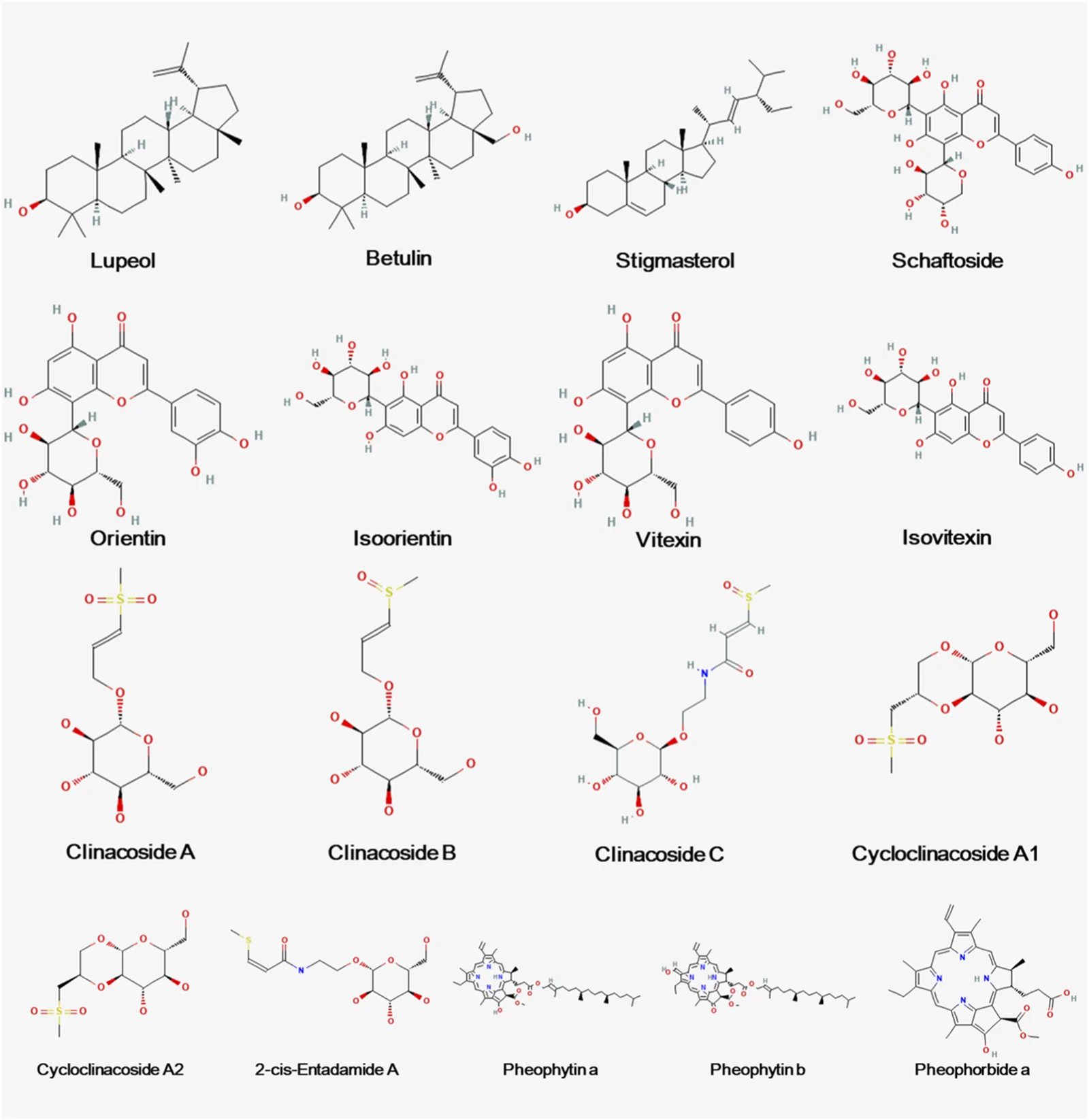
Representative chemical structures of major bioactive metabolites isolated from *Clinacanthus nutans*. The figure illustrates six categories of phytochemicals: triterpenoids (Lupeol, Betulin), steroidal metabolites (Stigmasterol), flavonoids (Schaftoside, Orientin, Isoorientin, Vitexin, Isovitexin), sulfur containing glycosides (Clinacoside A–C, Cycloclinacoside A1–A2, 2 cis Entadamide A), and chlorophyll related metabolites (Pheophytin a, Pheophytin b, Pheophorbide a). Chemical formula and structural information were obtained from PubChem database.

Over the past decade, scientific interest in this species has grown substantially, driven by consumer demand for complementary and alternative medicines and by evidence that multi-target natural products may overcome the limitations of single-target chemotherapeutics. This reflects a broader global trend toward natural product-based drug discovery and evidence-based herbal medicine, positioning *C. nutans* as a representative example within multi-target therapeutic research. Modern pharmacological studies have identified more than 130 metabolites, including triterpenoids, steroids, flavonoids, alkaloids, sulfur-containing metabolites, and glycosides (Figure 2) (Chia et al., 2021).

Among these, flavonoids and triterpenoids are particularly well studied, demonstrating antioxidant, anti-inflammatory, antiviral, antitumor, lipid-lowering, and hypoglycemic activities (Liew et al., 2024). Nevertheless, current evidence remains fragmented, largely confined to *in vitro* assays and small-scale animal studies, and lacks systematic evaluation of translational potential. This limitation highlights the need for a more integrated and critical synthesis of existing findings.

Unlike previous reports that focus narrowly on isolated pharmacological activities or specific metabolite classes, this review provides a comprehensive, evidence-stratified synthesis of phytochemistry, pharmacological mechanisms, and translational relevance. By critically assessing current knowledge, identifying gaps, and outlining future research priorities, it establishes a clear rationale for advancing the clinical and veterinary development of *C. nutans*. This integrated perspective distinguishes the present review from earlier fragmented accounts and offers an actionable roadmap for academic researchers and drug developers.

## 2 Literature search strategy

We conducted a comprehensive electronic search across multiple databases, including PubMed, Web of Science, and Google Scholar, using the keywords “*Clinacanthus nutans*,” “ethnomedicine,” “phytometabolites,” “anticancer,” “anti-inflammatory,” and “antiviral.” In addition, unpublished materials such as doctoral and M. Sc. theses, conference proceedings, and ethnobotanical textbooks were screened and selectively included. Inclusion criteria comprised original research articles with defined study designs, peer-reviewed publication type, English language, and direct relevance to *C. nutans*. Exclusion criteria included non-peer-reviewed sources and conference abstracts without full data. Patent databases (e.g., WIPO, Google Patents) were surveyed to capture translational developments related to *C. nutans*.

## 3 Taxonomic position and botanical characteristics

**FIGURE 3:**
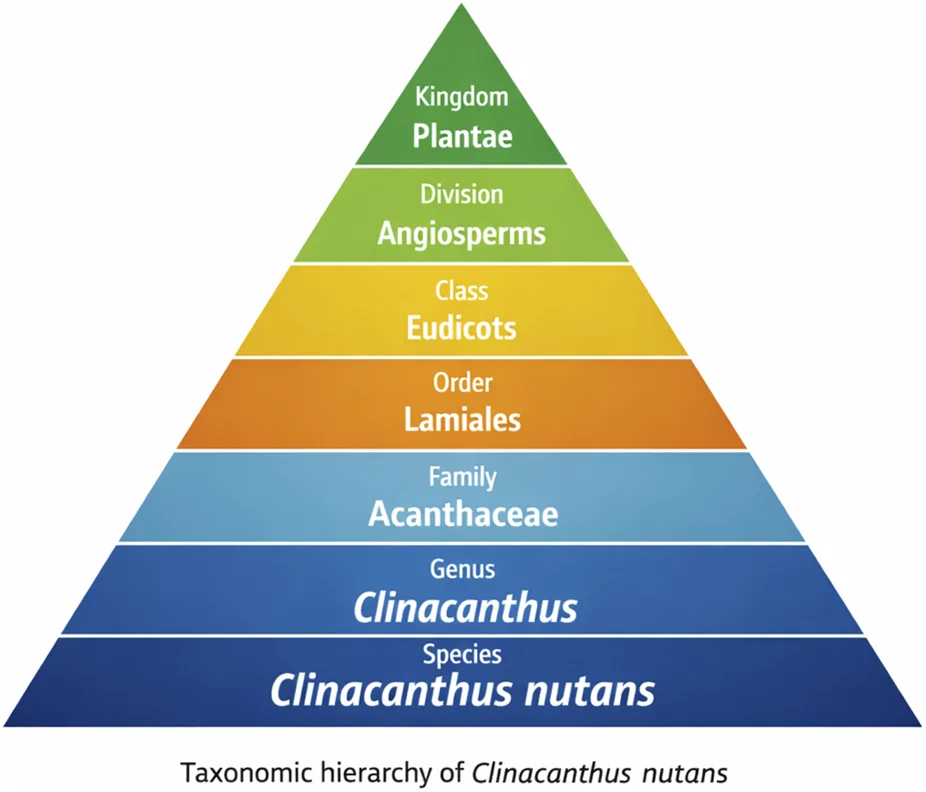
Taxonomic position and distribution of *Clinacanthus nutans* within the family Acanthaceae and order Lamiales.

*Clinacanthus nutans* is a perennial herb or shrub belonging to the family Acanthaceae and classified within the order Lamiales (Figure 3). It is native to southern China (Yunnan, Guangdong, Hainan, Taiwan) and widely distributed across Southeast Asia, including Thailand, Malaysia, Indonesia, Vietnam, Laos, Myanmar, and Cambodia. Plants typically attain a height of 1–3 m, with slender, erect or occasionally climbing cylindrical stems that become yellowish when dry and exhibit fine longitudinal striations.

**FIGURE 4:**
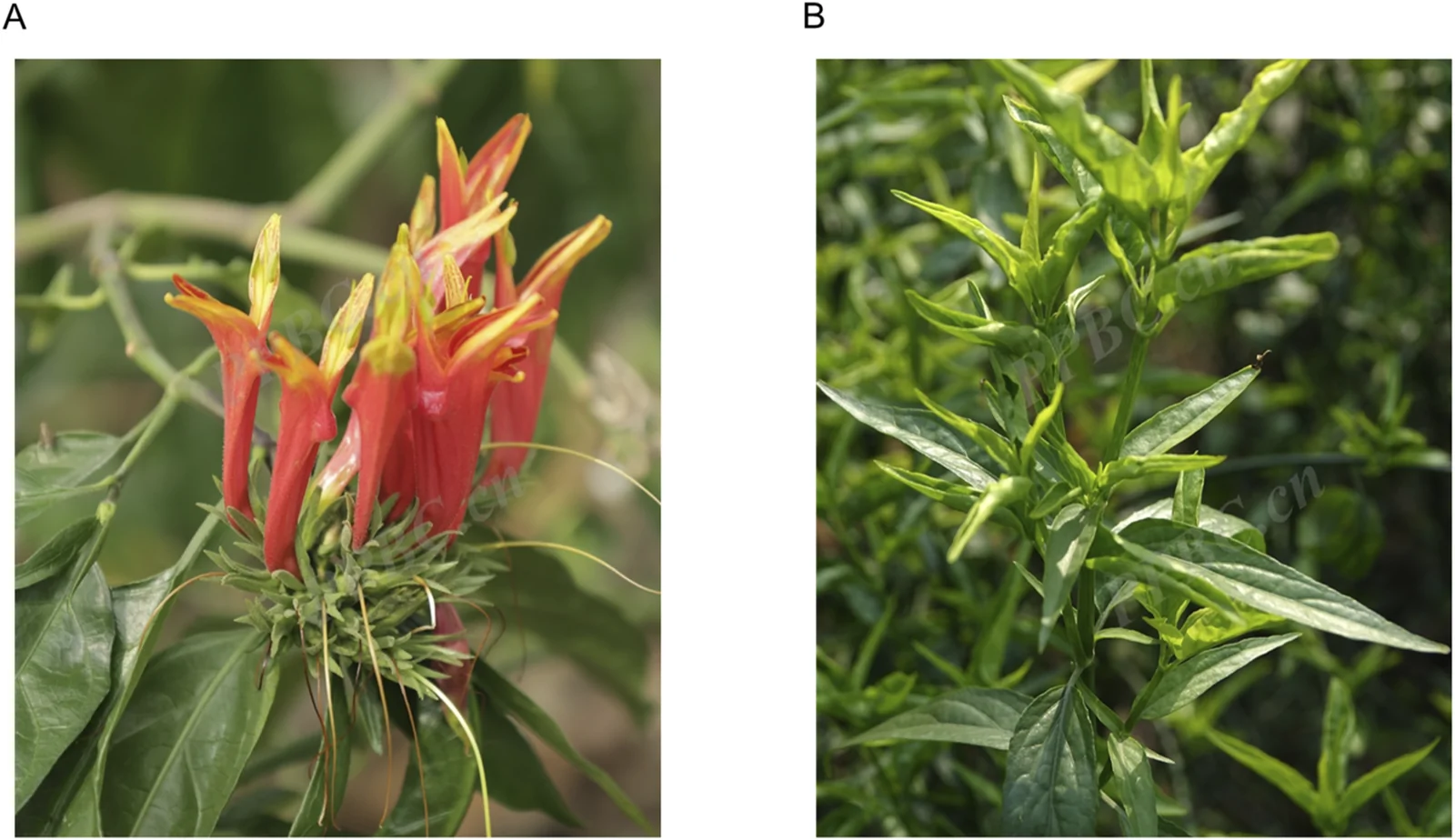
Morphological features of *Clinacanthus nutans*. **(A)** Flower. **(B)** Leaf.

Leaves are papery, lanceolate to ovate-lanceolate, measuring 5–11 cm in length and 1–4 cm in width, with caudate-acuminate apices, slightly oblique bases, entire margins, and pinnate venation bearing 5–6 pairs of lateral veins. Petioles are generally 5–7 mm or longer. Inflorescences occur as short racemes (∼1.5 cm), bearing linear bracts (∼8 mm) and reddish-purple tubular bilabiate corollas up to 4 cm long, externally pubescent. Sepals are ∼8 mm and gradually acuminate, while stamens and pistils are glabrous. Fruits are linear capsules containing numerous small seeds, although mature capsules are rarely observed. Flowering predominantly occurs in spring and summer (Figure 4) (China).

Ecologically, *C. nutans* thrives in tropical and subtropical climates under warm, humid conditions. It is commonly found along forest margins, secondary scrublands, and open lowland habitats, but also extends into mid-elevation regions. The species prefers well-drained sandy or loamy soils and requires abundant sunlight and seasonal rainfall for optimal growth. Environmental factors such as temperature, precipitation, and soil fertility strongly influence its distribution and abundance across different geographic regions.

## 4 Ethnomedicinal uses

A thorough understanding of the ethnomedicinal uses of *C. nutans* provides essential context for prioritizing pharmacological investigation. Across different regions, the plant has traditionally been indicated for skin ailments, snake bites, metabolic disorders, and inflammatory conditions, among others. Table 1 summarizes its documented traditional applications by geographic region and biological target, cross-referenced with the metabolite classes likely to mediate each activity. It is noteworthy that many of these ethnomedicinal claims, particularly anticancer and antiviral applications, have now received varying degrees of experimental corroboration, validating the merit of continued systematic investigation.

**TABLE 1:** Ethnomedicinal uses of *Clinacanthus nutans* by geographic region, target condition, and putative active class.

Region	Traditional use	Plant part	Preparation	Putative active class
Malaysia^1–4^	Cancer adjuvant therapy, herpes	Leaves	Juice, decoction	Flavonoids, sulfur glycosides
Thailand^5^ ^,^ ^6^	Herpes simplex, inflammation	Leaves	Topical extract	Flavonoids, triterpenoids
Indonesia^7^	Snake bites, skin infections	Leaves, stems	Poultice	Alkaloids, triterpenoids
China (Guangxi)^8^	Rheumatism, traumatic injury	Whole aerial parts	Decoction	Steroids, triterpenoids
China (Yunnan, Dai)^9^	Hepatitis, dampness-heat syndromes	Leaves	Boiled extract	Flavonoids, chlorophyll derivatives
Veterinary (emerging)^10^	Mastitis, wound healing	Leaves	Topical/oral extract	Steroids, sulfur glycosides

Reference:

1 Yang et al. (2013)

2 Goonasakaran (2013)

3 Sekar and Rashid (2016)

4 Rahim et al. (2016)

5 Keawpradub and Purintrapiban (2009)

6 Dampawan et al. (1977)

7 Nurulita et al. (2008)

8 Lin et al. (2023)

9 Fu and Sang (2024)

10 Panya et al. (2020)

## 5 Phytochemical metabolites

### 5.1 Triterpenoids

Triterpenoids are natural organic metabolites composed of six isoprene units and are widely distributed in plants. They possess multiple biological activities, including anti-inflammatory, immunomodulatory, and antitumor effects. Thai scholar Dampawan (1976) was the first to purify stigmasterol, lupeol, and β-sitosterol from the stems, leaves, and roots of *C. nutans*. In 1983, Lin et al. (1983) isolated lupeol, betulin, and β-sitosterol from the flowers of *C. nutans*. Lupeol, a chemopreventive agent, has been shown to inhibit various tumor cells, including lung, liver, colon, and osteosarcoma cells. Its mechanisms involve suppressing tumor cell proliferation, migration, and invasion, inducing cell-cycle arrest, and promoting apoptosis (AlMousa et al., 2025). In addition, studies have demonstrated that lupeol can protect against cardiotoxicity and hepatotoxicity induced by chemotherapeutic agents (Li et al., 2022; Singha et al., 2025). Pfarr et al. reported that betulin can enhance antitumor immune responses by stimulating immune organs, promoting T-lymphocyte function, and increasing the cytotoxic activity of natural killer (NK) cells, thereby effectively inhibiting tumor cell growth (Tuli et al., 2021). Betulin and its derivatives play important roles in antitumor mechanisms by regulating Bcl-2 family proteins, promoting cytochrome c release, and activating caspase cascades, ultimately inducing tumor cell apoptosis. Moreover, betulin exhibits significant anti-inflammatory and anticancer activities and can modulate multiple signaling pathways, highlighting its multi-target potential in cancer prevention and therapy (Tuli et al., 2021).

### 5.2 Steroidal metabolites

Plant-derived steroidal metabolites include steroidal saponins, phytosterols, and cardiac glycosides. Phytosterols are natural bioactive substances with structural similarity to cholesterol, mainly including sitosterol, stigmasterol, and β-carotene glycosides. They have demonstrated therapeutic potential in lowering cholesterol levels, preventing atherosclerosis, exerting antioxidant effects, and inhibiting tumor cell proliferation (Shen et al., 2024; Park et al., 2025; Poudel et al., 2023; El Omari et al., 2024). Due to their structural resemblance to cholesterol, phytosterols can competitively inhibit intestinal cholesterol absorption, thereby reducing blood lipid levels, and they also exhibit anti-inflammatory and anticancer potential (Shen et al., 2024; Nattagh-Eshtivani et al., 2022). Asudas et al. (2025) isolated stigmasterol and β-sitosterol from *C. nutans*.

Current research on phytosterols primarily focuses on their antitumor properties, including inhibition of carcinogen formation, suppression of tumor cell growth and proliferation, inhibition of angiogenesis, and modulation of tumor-related immune responses (Ahmad et al., 2025). Studies have shown that β-sitosterol can enhance the activity of various antioxidant enzymes, thereby increasing the resistance of phorbol 12-myristate 13-acetate (PMA)-induced macrophages to oxidative stress (Arivarasu, 2023). Research on stigmasterol has demonstrated that it exerts anti-melanoma effects by reducing reactive oxygen species (ROS) levels and regulating PD-L1 expression in melanoma cells, ultimately inducing apoptosis (Han et al., 2024). Recent studies further revealed that stigmasterol can alleviate lipopolysaccharide-induced mastitis in dairy cows by inhibiting inflammatory responses, suppressing ferroptosis, and maintaining the integrity of the blood–milk barrier (Sun et al., 2025). In addition, phytosterols have been shown to stimulate lymphocyte proliferation in peripheral blood (Shen et al., 2024), enhancing NK-cell cytotoxicity during the proliferative phase and thereby promoting immune responses. Mutakin et al. focused on the anticancer effects of β-sitosterol and its derivatives against breast cancer. Using both *in silico* and *in vitro* approaches, they primarily investigated its antiproliferative activity and apoptosis-inducing mechanisms. (Mutakin et al., 2025). Numerous studies have demonstrated that β-sitosterol and stigmasterol not only enhance antioxidant enzyme activity and reduce oxidative damage but also downregulate pro-inflammatory cytokines and modulate macrophage and lymphocyte functions, producing immunoregulatory effects in inflammatory models (Bakrim et al., 2022; Fan et al., 2023). Phytosterols (including β-sitosterol and stigmasterol) can significantly inhibit lipopolysaccharide-induced inflammatory responses in RAW264.7 macrophages by reducing key inflammatory mediators (NO, PGE2) and cytokine production (Yuan et al., 2019).

### 5.3 Flavonoids

Flavonoids, also known as plant flavones, are an important group of natural polyphenolic metabolites widely found in fruits, vegetables, tea, grains, and various medicinal plants. In 2024, Sabindo et al. (2024) isolated and purified six C-glycosyl flavonoids—schaftoside, isoorientin-7-O-β-D-glucopyranoside, orientin, isoorientin, vitexin, and isovitexin—from the n-butanol fraction and methanol extract of *C. nutans*. C-glycosyl flavonoids exhibit multiple biological activities, including antioxidant effects, regulation of glucose homeostasis, hepatoprotection, and antiviral activity (Xie et al., 2022; Mori et al., 2021). In addition, C-glycosyl flavonoids can reduce intracellular calcium levels, inhibit reactive oxygen species (ROS) generation, suppress apoptosis, and attenuate inflammatory responses, thereby protecting against myocardial ischemic injury (Verma et al., 2024). Several studies have demonstrated that C-glycosyl flavonoids exert inhibitory effects on tumor cell proliferation. Pacifico et al. (2010) isolated isoorientin from the roots and leaves of *Petrorhagia velutina* and found that it significantly inhibited the proliferation of HepG2 hepatoblastoma cells within 48 h. Both *in vitro* and *in vivo* studies have shown that certain C-glycosyl flavonoids (such as orientin, isoorientin, vitexin, and others) can activate the Nrf2-ARE signaling pathway, upregulate downstream antioxidant enzymes (SOD, CAT, GPx), reduce oxidative stress, stabilize mitochondrial membrane potential, and inhibit apoptosis pathways (Caspase-3/9) and intracellular calcium overload, thereby protecting against myocardial ischemia-reperfusion injury and hepatocellular damage (Fahmy et al., 2025; Yan et al., 2025). Yang and He (2022) reported that C-glycosyl flavonoids can also suppress the NF-κB signaling pathway and downregulate pro-inflammatory cytokines (TNF-α, IL-6, IL-1β), thereby alleviating inflammatory responses in various hepatotoxicity and metabolic disease models and improving the tissue repair microenvironment. Other studies have shown that certain C-glycosyl flavonoids exert antiviral effects by inhibiting viral replication and virus-induced inflammatory responses. Metabolites such as vitexin and puerarin have been shown to interfere with viral entry, inhibit viral protease activity, and modulate NF-κB–mediated inflammatory pathways, thereby exerting dual antiviral and anti-inflammatory effects (Al Azzahra and Muchtaridi, 2024). In addition, C-glycosyl flavonoids have been shown to induce cell-cycle arrest, activate mitochondrial-dependent apoptosis, and inhibit migration and invasion-related proteins, demonstrating antiproliferative and antimetastatic potential against tumor cells (Mohammad et al., 2025).

### 5.4 Alkaloids

Alkaloids are an important class of natural metabolites characterized by diverse structures and can be categorized into organic amines, pyrrolidines, pyridines, and other subclasses. They exhibit a wide range of biological activities, including anti-inflammatory, antioxidant, and antitumor effects (Amin et al., 2026). In 2019, Diao et al. (2019) first isolated one new furofuran lignan and eight alkaloids from the ethanol extract of the aerial parts of *C. nutans*. In recent years, plant-derived alkaloids have become a research hotspot in natural product chemistry due to their structural diversity and significant bioactivities. Studies have shown that certain alkaloids possess inhibitory effects on the replication of bacteria or specific viruses, demonstrating antibacterial and antiviral potential, and may provide auxiliary therapeutic benefits when administered during early infection or via local application (Faisal et al., 2023). Choudry et al. (2025) systematically summarized the structural elucidation and pharmacological activities of naturally occurring alkaloids over the past 5 years, emphasizing their potential in anti-inflammatory, antioxidant, and antitumor applications. Leclerc and Fournier further highlighted the central role of alkaloids in natural product chemistry, noting that their chemical properties and classification provide an important foundation for pharmacological research (Leclerc and Fournier, 2024). Bufo discussed the dual nature of alkaloids from toxicological and pharmacological perspectives, noting that while some alkaloids exhibit toxicity, they may also serve as valuable lead metabolites for drug development, underscoring their significance in medicinal chemistry (Adamski et al., 2020).

### 5.5 Sulfur-containing glycosides

Sulfur-containing glycosides are a class of metabolites in which the glycosidic bond contains a sulfur atom instead of oxygen. These metabolites exhibit various biological activities, including anticancer, antioxidant, antidiabetic, anti-inflammatory, immunomodulatory, antibacterial, and antiallergic effects (Sachdeva et al., 2022). Teshima et al. (1998) was the first to isolate five novel sulfur-containing glycosides from the leaves of *C. nutans*, with molecular formulas C_10_H_18_O_8_S, C_10_H_18_O_7_S, C_10_H_21_NO_8_S, C_10_H_18_O_8_S, and C_10_H_18_O_8_S, which were named clinacoside A, clinacoside B, clinacoside C, cycloclinacoside A1, and cycloclinacoside A2, respectively. Tu et al. (2014) later isolated four additional sulfur-containing glycosides from *C. nutans*, namely, clinamide A, clinamide B, clinamide C, and 2-cis-entadamide A. *In vitro* studies have shown that clinacoside/clinamide metabolites possess free-radical-scavenging activity, inhibit lipid peroxidation, and protect mitochondrial function. Their mechanisms may involve upregulation of antioxidant enzymes downstream of the Nrf2-ARE signaling pathway or direct elimination of reactive oxygen species (Ng et al., 2022). In inflammatory cell models, sulfur-containing glycosides can downregulate pro-inflammatory cytokines (such as TNF-α and IL-6) and inflammation-related enzymes (such as iNOS and COX-2), while inhibiting NF-κB signaling and modulating macrophage phenotypes (Bahiraii et al., 2022). Active hydrolysis products of sulfur-containing glycosides (such as sulforaphane) have also been shown to improve glucose tolerance and enhance insulin signaling, potentially through antioxidant and anti-inflammatory mechanisms that protect pancreatic islets and peripheral tissues (Mohamadi et al., 2023; Alves et al., 2025).

### 5.6 Chlorophyll-related metabolites

Chlorophylls possess antioxidant, anti-inflammatory, detoxifying, toxin-binding, and tissue-repair-promoting activities. The leaves of *C. nutans* contain multiple chlorophyll-related substances. In 2001, Ayudhya et al. isolated and identified two pheophytins—pheophytin a and pheophytin b—from the chloroform crude extract of *C. nutans* (Ayudhya et al., 2001). In 2006, Sakdarat et al. further isolated and identified eight chlorophyll derivatives from *C. nutans*, three of which exhibited structures similar to chlorophyll a and b, while the structures of the remaining five metabolites had not yet been elucidated (Santi et al., 2006). Moreover, pheophorbide a was found to induce apoptosis in human hepatocellular carcinoma cells without exerting toxic effects on normal human hepatocytes (Fakudze et al., 2025). Studies have also demonstrated that two pheophytin derivatives extracted from *Chaetomorpha brachygona* can inhibit tumor necrosis factor-α (TNF-α)-induced activation of the nuclear factor-κB (NF-κB) signaling pathway in human cervical cancer cells (Haq et al., 2019).

### 5.7 Other metabolites

In addition, *C. nutans* contains various amino acids and trace elements, which are closely associated with its anti-inflammatory, antioxidant, and cardiovascular-protective activities (Ashraf, 2019). Plant-derived amino acids have been shown to regulate metabolic and immune functions and may help prevent metabolic disorders such as obesity and diabetes (Trovato et al., 2021; Jain and Goomer, 2020). Trace elements such as zinc, iron, and copper are essential cofactors for numerous enzymes and antioxidant systems, supporting cellular metabolism and immune defense. While appropriate intake is beneficial to health, excessive amounts may lead to toxicity (Kaur and Srivastava, 2023; Prasad, 2025). A comprehensive summary of identified phytochemical metabolites, their metabolite classes, and associated bioactivities is presented in Table 2.

**TABLE 2:** Representative phytochemical metabolites of *Clinacanthus* nutans, their metabolite class, and reported biological activities.

Metabolite	Class	Key biological activities	References
Lupeol	Triterpenoid	Antitumor, cardioprotective, hepatoprotective	(AlMousa et al., 2025; Li et al., 2022; Singha et al., 2025)
Betulin	Triterpenoid	Antitumor, immunostimulatory, anti-inflammatory	(Tuli et al., 2021)
β-Sitosterol	Phytosterol	Lipid-lowering, antioxidant, immunomodulatory	(Ahmad et al., 2025; Bakrim et al., 2022)
Stigmasterol	Phytosterol	Anti-melanoma, anti-inflammatory, anti-mastitis	(Han et al., 2024; Sun et al., 2025)
Orientin	C-glycosyl flavonoid	Antioxidant, cardioprotective, antiviral	(Fahmy et al., 2025; Al Azzahra and Muchtaridi, 2024)
Isoorientin	C-glycosyl flavonoid	Hepatoprotective, antitumor	(Pacifico et al., 2010; Yan et al., 2025)
Vitexin	C-glycosyl flavonoid	Antiviral, anti-inflammatory	(Al Azzahra and Muchtaridi, 2024)
Schaftoside	C-glycosyl flavonoid	Antioxidant, anti-inflammatory	(Teshima et al., 1997)
Clinacoside A – C	Sulfur glycoside	Antioxidant, anti-inflammatory	(Teshima et al., 1998; Ng et al., 2022)
Clinamide A – C	Sulfur glycoside	Antioxidant, antidiabetic	(Tu et al., 2014)
Pheophytin a/b	Chlorophyll derivative	Antitumor, NF-κB inhibition	(Ayudhya et al., 2001; Huang et al., 2007)
Pheophorbide a	Chlorophyll derivative	Cytotoxic (cancer cells), pro-apoptotic	(Cheng et al., 2001; Chan et al., 2006)

## 6 Pharmacological activities of *Clinacanthus nutans*

**FIGURE 5:**
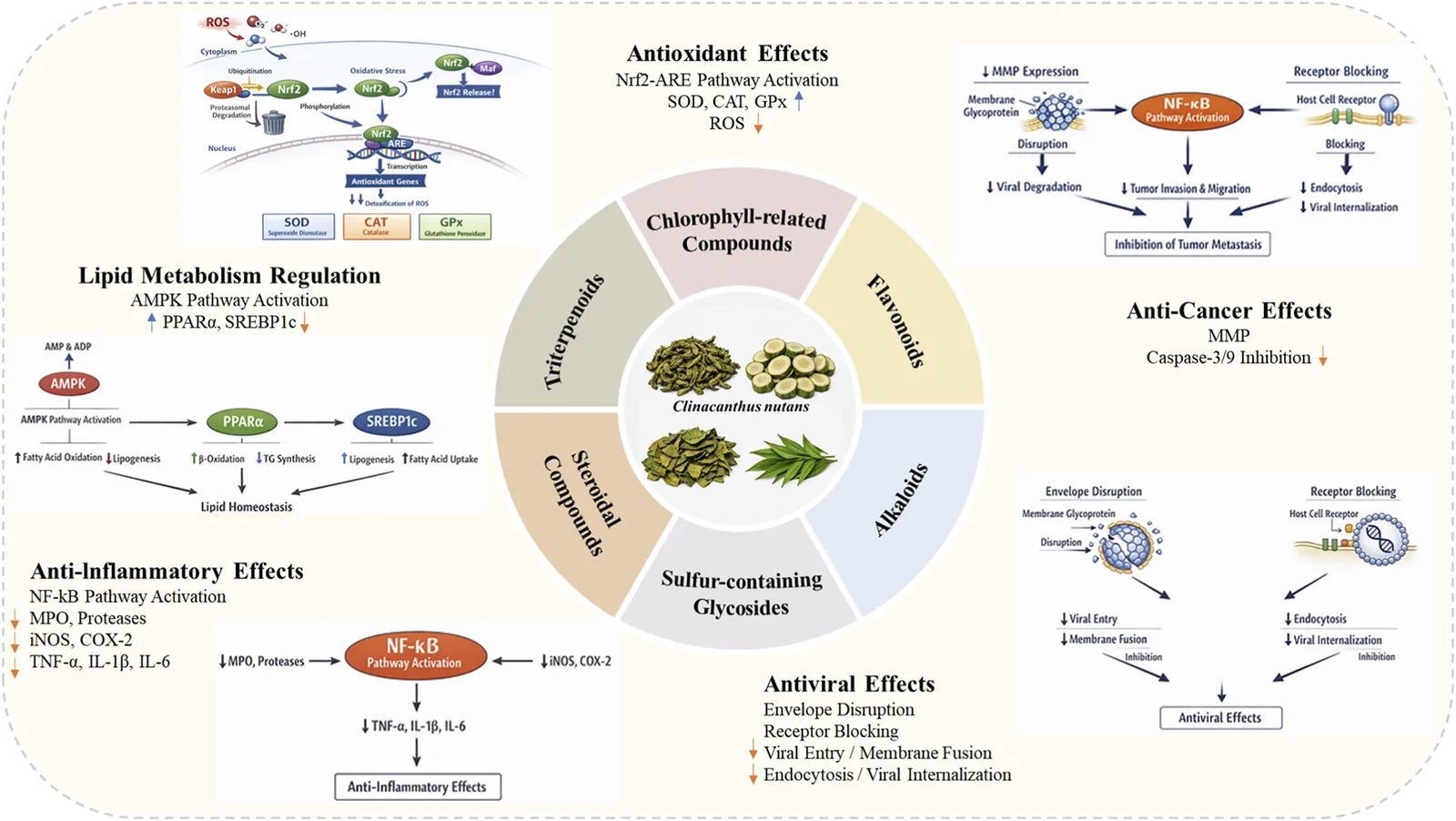
Mechanism based illustration summarizing the major bioactive metabolites and pharmacological targets of *Clinacanthus nutans*. The central panel highlights the principal phytochemical groups—triterpenoids, flavonoids, steroidal metabolites, alkaloids, and sulfur containing glycosides—while the surrounding modules depict their associated biological effects and signaling pathways, including Nrf2 ARE (antioxidant), NF κB (anti inflammatory and anti cancer), AMPK (lipid metabolism regulation), and viral envelope/receptor interactions (antiviral).

**FIGURE 6:**
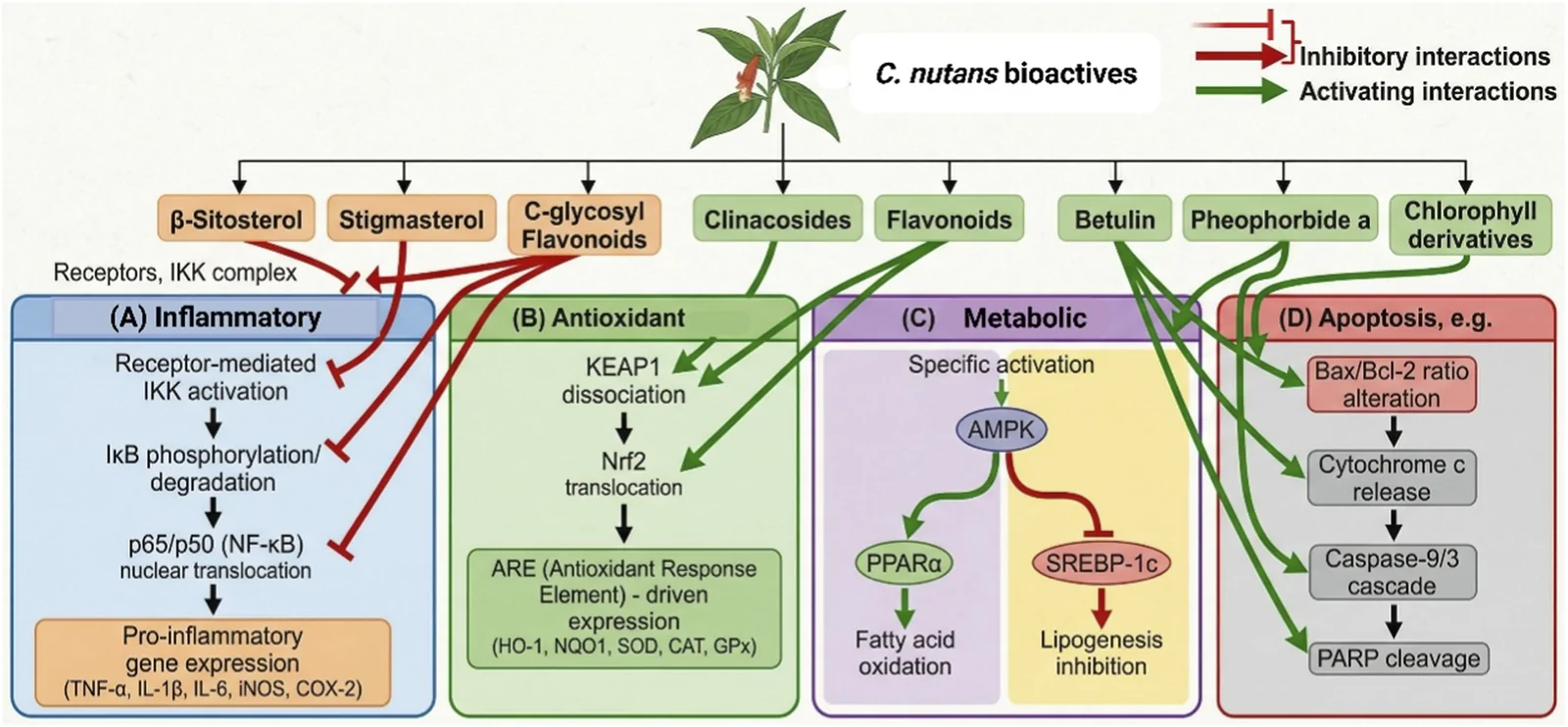
Integrated signaling network mediating the pharmacological activities of *C. nutans* metabolites. The four primary axes depicted are: **(A)** the NF-κB inflammatory axis (from receptor-mediated IKK activation → IκB phosphorylation/degradation → p65/p50 nuclear translocation → pro-inflammatory gene expression of TNF-α, IL-1β, IL-6, iNOS, COX-2), indicating points of inhibition by β-sitosterol, stigmasterol, and C-glycosyl flavonoids; **(B)** the Nrf2/ARE antioxidant axis (KEAP1 dissociation → Nrf2 nuclear translocation → ARE-driven expression of HO-1, NQO1, SOD, CAT, GPx), activated by clinacosides and flavonoids; **(C)** the AMPK/PPARα/SREBP-1c metabolic axis governing lipogenesis and fatty acid oxidation; and **(D)** the mitochondrial apoptosis axis (Bax/Bcl-2 ratio → cytochrome c release → caspase-9/3 cascade → PARP cleavage), activated by betulin, pheophorbide a, and chlorophyll derivatives in cancer cells.

The pharmacological profile of *C. nutans* is remarkably broad, encompassing antioxidant, anti-inflammatory, antiviral, anticancer, lipid-lowering, hypoglycemic, hepatoprotective, cardioprotective, immunomodulatory, and antibacterial activities. This multifaceted bioactivity is attributable to the structural diversity of its phytochemical constituents, which may act synergistically across multiple molecular targets and signaling networks. The following subsections critically review the evidence for each major pharmacological activity, highlighting key mechanistic insights and identifying gaps that remain to be addressed. Figure 5 offers an integrated overview of the principal signaling pathways mediating the broad pharmacological activities of *C. nutans*, while Figure 6 highlights the detailed relationships between its major bioactive metabolite classes and their corresponding pharmacological effects.

### 6.1 Antioxidant activity

Many active metabolites in *C. nutans* exhibit significant antioxidant and free-radical-scavenging capacities. Pannangpetch et al., (2007) investigated the antioxidant activity of the ethanolic extract of dried *C. nutans* leaves. The extract exhibited strong free radical scavenging activity in the DPPH assay, with a maximum inhibition of 67.65% ± 6.59% and an IC_50_ of 110.4 ± 6.59 μg/mL. It also demonstrated a FRAP value equivalent to 17 mg ascorbate/g extract, significantly suppressed peroxide production in PMA-stimulated rat macrophages, and protected erythrocytes against AAPH-induced hemolysis with an IC_50_ of 359.38 ± 14.02 μg/mL. Yong et al. (2013) evaluated the antioxidant and antiproliferative effects of *C. nutans* leaf extracts prepared with chloroform, methanol, and water. Antioxidant activity was assessed using DPPH, galvinoxyl, nitric oxide, and hydrogen peroxide radical scavenging assays, while antiproliferative activity was tested against HepG2, NCL-H23, SNU-1, HeLa, LS-174T, K562, and Raji cancer cell lines using the MTT assay. The chloroform extract showed the strongest activity against DPPH and galvinoxyl radicals, but weaker effects on nitric oxide and hydrogen peroxide. At 100 μg/mL, the chloroform extract inhibited proliferation of K-562 and Raji cells by 91.28% ± 0.03% and 88.97% ± 1.07%, respectively, and exerted concentration-dependent effects on other cancer cell lines, though not on IMR-32 cells. In recent years, domestic and international studies have identified numerous antioxidant metabolites in *C. nutans* and conducted a series of experimental investigations. Cellular and animal studies have confirmed that *C. nutans* and its active metabolites can activate the Nrf2-ARE signaling pathway, upregulate downstream antioxidant enzymes such as superoxide dismutase (SOD), catalase (CAT), and glutathione peroxidase (GPx), and thereby enhance endogenous antioxidant defenses (Ng et al., 2022, Nga et al., 2026). Meanwhile, the extract can inhibit NF-κB–mediated inflammatory responses and reduce the generation of inflammation-related reactive oxygen species (ROS) (Liew et al., 2024). At the cellular level, metabolites of *C. nutans* can stabilize mitochondrial membrane potential, alleviate intracellular Ca^2+^ overload, and suppress caspase-3/9-dependent apoptotic pathways, thereby establishing a multilayered protective mechanism ranging from free-radical scavenging to cellular protection (Teoh, 2021). Collectively, these findings demonstrate that *C. nutans* exerts protective effects against oxidative stress–related tissue injury by coordinately regulating oxidative stress and inflammatory signaling pathways.

### 6.2 Anti-inflammatory activity

Wanikiat et al. (2008) investigated the anti-inflammatory activities of *C. nutans* extracts using carrageenan-induced paw edema and ethylphenylpropiolate-induced ear edema models in rats. The extracts produced potent, dose-dependent inhibition of edema formation and significantly reduced MPO activity in inflamed tissues. In human neutrophils, the extracts concentration-dependently inhibited fMLP-induced chemotaxis, superoxide anion generation, and MPO and elastase release, without affecting cell viability or apoptosis. These findings suggest that the anti-inflammatory properties of *C. nutans* are mediated, at least in part, by inhibition of neutrophil responsiveness. Furthermore, studies have shown that *C. nutans* can interfere with downstream inflammatory signaling pathways by inhibiting NF-κB activation and nuclear translocation, downregulating the expression of iNOS and COX-2, and reducing the secretion of key pro-inflammatory cytokines such as TNF-α, IL-1β, and IL-6. Through these mechanisms, *C. nutans* effectively suppresses the amplification of inflammatory cascades at the molecular level (Ong et al., 2022). These findings collectively position *C. nutans* as a broad-spectrum anti-inflammatory agent acting at multiple nodes within the inflammatory cascade, a profile of particular relevance for chronic inflammatory diseases where monotherapy with single-target agents frequently proves insufficient.

### 6.3 Antiviral activity

*Clinacanthus nutans* is a well-known medicinal plant in Southeast Asian countries, particularly Thailand, and has long been used for treating various diseases. Abd Wahab et al. (2021) reported the *in vitro* antiviral activity of methanolic crude extracts of *C. nutans* against HSV-1, with a CC_50_ value of 1.625 mg/mL in Vero cells and selective indices of 11, 6, and 3 in post-treatment, pre-treatment, and virucidal assays, respectively. These findings highlight a consistent theme: the antiviral activity of *C. nutans* predominantly operates at pre-entry and entry stages of the viral lifecycle, a mechanism with clinical promise for prophylactic or topical applications, and one that warrants investigation against clinically relevant emerging viruses.

### 6.4 Anticancer activity

Multiple active metabolites of *C. nutans* exhibit antitumor properties; however, current research in this area remains relatively limited. A bioactive fraction (CNM.CF5.5) isolated from *C. nutans* leaves demonstrated potent anticancer activity. Cytotoxicity assays revealed significant growth inhibition of MCF-7 and A549 cells, with IC_50_ values of 61.67 μg/mL and 81.62 μg/mL, respectively. HR-LCMS profiling identified diverse phytochemicals, and molecular docking suggested that vitexin and isovitexin strongly interact with cancer-associated proteins such as CDK1, CDK2, and NQO1. These findings support the potential of *C. nutans* bioactives as promising candidates for cancer therapy. (Jahagirdar et al., 2025). Lin et al. (2021) reported that the chloroform fraction of *C. nutans* exhibited the most potent antiproliferative activity on human erythroleukemia K-562 cells and lymphoma Raji cells, while also showing dose-dependent inhibition of hepatocellular carcinoma, lung cancer, gastric cancer, cervical cancer, and colon cancer cell lines. An ethyl acetate fraction of *C. nutans* (CNEAF) exhibited potent cytotoxicity against HCT116 colorectal cancer cells (IC_50_ = 48.81 μg/mL). Mechanistic studies revealed that CNEAF induced apoptosis through mitochondrial membrane potential dissipation, Bax/Bak upregulation, Bcl-2/Bcl-xL downregulation, and activation of caspase-3, -9, -8, and -10. Upregulation of death receptor 5 suggested involvement of both intrinsic and extrinsic apoptotic pathways. In addition, autophagy was evidenced by LC-3 accumulation and p62 degradation. Importantly, the apoptosis and autophagy were ROS-dependent, as confirmed by reversal with N-acetylcysteine. These findings highlight ROS-mediated apoptosis and autophagy as key anticancer mechanisms of *C. nutans*. (Wang et al., 2019). Recent studies have highlighted the anticancer potential of *C. nutans* bioactive metabolites against breast cancer. Isolated metabolites such as β-sitosterol, stigmasterol, shaftoside, and lupeol demonstrated significant cytotoxicity toward MCF-7 and MDA-MB-231 cells, inducing apoptosis through cell cycle arrest, mitochondrial disruption, caspase activation, and modulation of ROS levels. Notably, these metabolites exhibited synergistic effects when combined with conventional chemotherapeutics including tamoxifen, sorafenib, doxorubicin, and 5-fluorouracil, underscoring their promise as adjunctive agents in breast cancer therapy (Asudas et al., 2025). Notably, the multi-target nature of its metabolite metabolites—simultaneously engaging apoptotic, antioxidant, anti-angiogenic, and anti-metastatic pathways—represents a compelling rationale for studying *C. nutans* as a combination partner or chemosensitizer in established oncology regimens. Future work should utilize patient-derived organoid and xenograft models to more faithfully replicate clinical cancer heterogeneity.

### 6.5 Lipid-lowering and hypoglycemic effects

In a cholesterol-supplemented western diet model of metabolic dysfunction-associated steatohepatitis (MASH), ethanolic extracts of *C. nutans* leaves significantly attenuated disease progression in male C57BL/6 mice. Treatment at doses of 100–400 mg/kg reduced liver-to-body weight ratio, NAFLD activity scores, and histological features such as lobular inflammation. Serum AST, ALT, and hepatic malondialdehyde levels were markedly lowered, with effects surpassing those of atorvastatin, although lipid profile (LDL/HDL ratio) remained unaffected. These findings suggest that *C. nutans* exerts hepatoprotective effects primarily through antioxidative mechanisms attributable to its high tannin content (Hasanatuludhhiyah et al., 2025). Metabolomics and molecular docking studies have identified key phytometabolites in C. nutans leaves contributing to hepatoprotective and antioxidative effects. Hexane and ethyl acetate fractions reduced ROS levels in HepG2 cells by 36% and 27.6%, respectively, and significantly increased glutathione levels. Clinacoside B, clinacoside C, and isoschaftoside were detected, with isoschaftoside showing strong binding to Keap1, suggesting modulation of the Keap1-Nrf2 pathway. These findings highlight isoschaftoside as a promising candidate for managing chronic liver conditions such as non-alcoholic fatty liver disease (Nga et al., 2026). Based on chemical composition analysis, network pharmacology predictions, and *in vivo* experimental validation, the flavonoids, sterols, and sulfur-containing glycosides in *C. nutans* may exert lipid-lowering and hepatoprotective effects by activating the AMPK signaling pathway, upregulating PPARα, downregulating SREBP-1c, and inhibiting NF-κB–mediated inflammatory signaling. Through these mechanisms, *C. nutans* promotes fatty acid β-oxidation, suppresses lipogenesis, and reduces hepatic inflammation (Teoh, 2021; Liew et al., 2024; Fu and Sang, 2024). These findings carry significant clinical relevance given the global burden of non-alcoholic fatty liver disease (NAFLD) and metabolic syndrome, for which safe, orally bioavailable plant-derived agents are urgently needed.

### 6.6 Other activities

Extracts and fractions of *C. nutans* have demonstrated broad antimicrobial activity. Petroleum ether, ethyl acetate, and methanol extracts inhibited *Bacillus cereus*, *Escherichia coli*, *Salmonella Typhimurium*, and *Candida albicans*, with Fraction 7 from the ethyl acetate extract showing MIC/MBC values of 1.39 mg/mL against *B. cereus* and *C. albicans*, comparable to ampicillin and amphotericin B. These findings highlight the potential of *C. nutans* bioactives as natural antimicrobial agents with both antibacterial and antifungal properties (Arullappan et al., 2014). Overall, the broad-spectrum antimicrobial efficacy of *C. nutans* extracts, with activity comparable to standard antibiotics and antifungals, underscores its potential as a natural source of therapeutic agents for both bacterial and fungal infections.

## 7 Evidence stratification and translational relevance

A critical appraisal of the current literature on *C. nutans* reveals that evidence is unevenly distributed across experimental tiers, as summarized in Table 3. *In vitro* studies have provided important mechanistic insights, including antioxidant, anti-inflammatory, antiviral, and anticancer activities, yet these assays often oversimplify biological complexity and lack systemic relevance. Animal studies have demonstrated pharmacodynamic effects in models of inflammation, metabolic dysfunction, and cancer, but species differences, short study durations, and limited reproducibility constrain their predictive value for human applications. Clinical studies remain scarce, with only preliminary reports of antiviral and metabolic benefits, and no rigorous Phase I/II trials to substantiate therapeutic claims.

**TABLE 3:** Ethnomedicinal uses of *Clinacanthus nutans*, representative metabolites, corresponding scientific evidence, and validation status.

Traditional use	Representative metabolites	Scientific evidence (references)	Status
Cancer adjuvant, herpes	Orientin, Isoorientin, Clinacoside A–C	Antioxidant, antiviral (HSV), antitumor (Teshima et al., 1997; Teshima et al., 1998; Ng et al., 2022; Tu et al., 2014; Pacifico et al., 2010; Yan et al., 2025)	Validated
Herpes simplex, inflammation	Vitexin, Lupeol	Antiviral, anti-inflammatory; clinical use in herpes (Teshima et al., 1997; Al Azzahra and Muchtaridi, 2024; AlMousa et al., 2025; Singha et al., 2025)	Validated
Snake bites, skin infections	Betulin, Stigmasterol	Antitumor, anti-inflammatory; no systematic evidence for snake bites (Tuli et al., 2021; Han et al., 2024; Sun et al., 2025)	Insufficient
Rheumatism, traumatic injury	β-Sitosterol, Lupeol	Anti-inflammatory, immunomodulatory; lacking clinical validation (Ahmad et al., 2025; Bakrim et al., 2022)	Insufficient
Hepatitis, dampness-heat syndromes	Isoorientin, Pheophytin a/b	Hepatoprotective, NF-κB inhibition; only *in vitro* evidence (Pacifico et al., 2010; Ayudhya et al., 2001; Huang et al., 2007; Yan et al., 2025)	Preliminary
Mastitis, wound healing	Stigmasterol, Clinamide A–C	Anti-mastitis, antioxidant, antidiabetic; limited animal data (Tu et al., 2014; Han et al., 2024; Sun et al., 2025)	Insufficient

In addition to experimental evidence, several patents have been reported covering extraction methods, pharmaceutical formulations, and therapeutic applications of *C. nutans*. These records highlight ongoing translational interest and provide a foundation for future development (Table 4).

**TABLE 4:** *Representative patents related to Clinacanthus nutans*, including applicant/inventor, year, application field, and patent number.

Applicant/Inventor	Year	Application field	Patent no.
Taiwan MOHW	2021–2023	Extraction; osteoporosis; neurodegeneration; wound healing	TW202322839A
Zhuhai Hopegenes Medical & Pharmaceutical Institute	2020	Functional food for pancreatic cancer	CN111602816A
Zhuhai Dahengqin Technology Development	2020	Anticancer functional food	CN111602816A
Patsnap Eureka	2016	*C. nutans* tea beverage formulation	CN105341244A
South China Agricultural University	2025	Hepatoprotective drug using *C. nutans* extract via MAPK-Nrf2 pathway	CN120053506A
Zhongshan Hospital Fudan University	2023	Ulcerative colitis treatment using *Clinacanthus nutans* extract	CN117414379A
Hainan Medical College	2023	Anti-heart failure drug using total flavonoids of *Clinacanthus nutans*	CN115844933A
Fuzhou University	2023	Anti-inflammatory and antioxidant polypeptide derived from *Clinacanthus nutans*	CN114989248A

Patent information was retrieved from Google Patents.

## 8 Key signaling pathways and molecular mechanisms

A defining feature of *C. nutans* that distinguishes it from single-metabolite drugs is its capacity to modulate multiple, interconnected signaling pathways simultaneously. Figure 6 provides an integrated map of the major pathways implicated in its pharmacological effects.

### 8.1 NF-κB pathway

The NF-κB signaling pathway is a central hub for inflammatory gene expression, regulating the production of cytokines (TNF-α, IL-1β, IL-6), adhesion molecules, and enzymes (iNOS, COX-2) (Roberti et al., 2022). Metabolites of *C. nutans*—notably β-sitosterol, stigmasterol, and C-glycosyl flavonoids—inhibit IκB kinase (IKK) activity, thereby preventing IκB phosphorylation and degradation, retaining NF-κB in an inactive cytoplasmic complex, and suppressing downstream inflammatory gene transcription (Yang and He, 2022; Ong et al., 2022). This mechanism underlies the documented anti-inflammatory and antitumor effects of the plant.

### 8.2 Nrf2/ARE pathway

The Nrf2/antioxidant response element (ARE) axis represents the primary endogenous defense against oxidative stress (Ngo and Duennwald, 2022). Activation of Nrf2 by *C. nutans* metabolites—especially clinacosides and C-glycosyl flavonoids—leads to dissociation from its repressor KEAP1, nuclear translocation, and transcriptional induction of cytoprotective genes including heme oxygenase-1 (HO-1), NAD(P)H quinone dehydrogenase 1 (NQO1), and the glutathione biosynthesis enzyme γ-GCS (Ng et al., 2022, Nga et al., 2026). This pathway also cross-talks with NF-κB, as Nrf2 activation can suppress NF-κB, creating a dual anti-inflammatory and antioxidant effect (Gao et al., 2022).

### 8.3 AMPK pathway

AMP-activated protein kinase (AMPK) serves as a master energy sensor and metabolic regulator (Trefts and Shaw, 2021). *Clinacanthus nutans* extracts appear to activate AMPK, which in turn upregulates PPARα (promoting fatty acid oxidation) and downregulates SREBP-1c (reducing lipogenesis), collectively attenuating hepatic steatosis (Liew et al., 2024; Fu and Sang, 2024). AMPK activation also suppresses mTORC1, which could contribute to the anti-proliferative effects observed in certain cancer cell lines (Smiles et al., 2024).

### 8.4 Apoptosis-related pathways

Multiple metabolites of *C. nutans* promote intrinsic (mitochondrial) apoptosis in cancer cells. This involves downregulation of anti-apoptotic Bcl-2 and Bcl-xL, upregulation of pro-apoptotic Bax and Bad, cytochrome c release into the cytoplasm, and sequential activation of caspase-9 and caspase-3 (Ismail et al., 2022). Betulin, pheophorbide a, and chlorophyll derivatives have each been independently shown to engage this pathway (Tuli et al., 2021; Cheng et al., 2001; Chan et al., 2006). Induction of G1 or G2/M cell cycle arrest—mediated by CDK inhibitor upregulation—has also been documented (Teoh, 2021; Wang et al., 2019).

## 9 Network pharmacology and systems biology perspective

Network pharmacology has emerged as a powerful computational tool for systematically predicting multi-metabolite, multi-target interactions in complex plant-based medicines (Joshi et al., 2025). Applied to *C. nutans,* network pharmacology analyses have confirmed that the major active metabolites (lupeol, β-sitosterol, stigmasterol, orientin, isoorientin) converge on core target proteins including TP53, AKT1, EGFR, TNF, IL-6, and PTGS2 (COX-2), which are central nodes in cancer, inflammation, and metabolic disease networks (Liew et al., 2024; Teoh, 2021).

Such analyses predict synergistic interactions among *C. nutans* metabolites that would not be predictable from single-metabolite studies alone, and provide a hypothesis-generating platform for future bioactivity-guided fractionation. Molecular docking studies have further corroborated direct binding interactions between orientin and isoorientin with NF-κB p65 and Nrf2 regulatory proteins, supporting their mechanistic roles (Fahmy et al., 2025). However, these computational predictions require rigorous experimental validation using target engagement assays and genetically modified cell lines before mechanistic conclusions can be drawn (Zhang et al., 2025).

## 10 Safety profile and toxicological considerations

A rigorous assessment of safety is indispensable before any botanical drug can advance to clinical use (Sethi et al., 2025). Early toxicological investigations reported that the oral LD_50_ of methanol leaf extract in mice exceeded 1.8 g/kg, with no mortality observed at doses up to 1.8 g/kg (Wen et al., 2012). Subsequent subacute studies in rats showed no adverse effects at doses up to 1 g/kg/day (P’ng et al., 2013). , while further evaluation confirmed good tolerability in mice at a single oral dose of 5,000 mg/kg, with a provisional NOAEL exceeding 2,500 mg/kg/day during 28-day subchronic administration (Zakaria et al., 2016). Later subchronic studies with doses exceeding 2 g/kg/day similarly did not report adverse effects, although detailed dose–response characterization and histopathological validation remain lacking (Monirvaghefi et al., 2023). Most recently, polysaccharide fractions (CNBP) administered orally to rats at doses up to 3,000 mg/kg/day caused no mortality or overt toxicity in acute and 14-day subacute studies, though mild hepatic effects were observed at ≥ 500 mg/kg/day, as indicated by elevated alkaline phosphatase levels (Chia et al., 2025). Importantly, no reproductive toxicity, genotoxicity, or teratogenicity data are currently available for *C. nutans* extracts or isolated metabolites, representing a critical gap for regulatory approval.

In addition, the absence of chronic toxicity, carcinogenicity, reproductive and developmental toxicity, and human safety data critically undermines the clinical translation of *C. nutans*. Without these datasets, long-term risk profiles remain undefined, regulatory approval pathways cannot be adequately addressed, and patient safety cannot be assured. Similarly, the lack of systematic herb–drug interaction studies poses a major barrier, as potential pharmacokinetic conflicts with conventional therapies may compromise efficacy or increase toxicity. These gaps collectively highlight that current toxicological evidence, while encouraging, is insufficient to support clinical application without further rigorous investigation.

Overall, current toxicological evidence indicates low acute and subacute toxicity of *C. nutans* extracts, but data remain limited. Rigorous studies addressing chronic toxicity, carcinogenicity, reproductive and genetic safety, and human safety are still required to support clinical translation.

## 11 Conclusion and future outlook

This review highlights the ethnomedicinal relevance, phytochemical diversity, and pharmacological potential of *Clinacanthus nutans*, underscoring its importance as a promising Southeast Asian medicinal plant. Current evidence supports antiviral, antioxidant, and anti-inflammatory activities, while antitumor findings remain preliminary. Key methodological limitations include incomplete dose range reporting, lack of minimum active concentration data, short study durations, absence of standardized controls, variability in extract preparation, and insufficient taxonomic authentication of plant material. In addition, critical toxicological gaps remain, including the absence of reproductive toxicity, genotoxicity, teratogenicity, and long-term safety data, which are essential for regulatory acceptance. These constraints reduce reproducibility and weaken the reliability of therapeutic claims for *C. nutans*.

Future research should focus on bioactivity-guided isolation and structural characterization of novel metabolites using advanced spectroscopic methods. Mechanistic *in vivo* studies with validated disease models are needed to confirm pathway interactions and clarify whether multi-target effects represent true synergy. Pharmacokinetic and metabolomic profiling of key bioactives (orientin, isoorientin, lupeol, clinacosides) is essential to establish bioavailability and metabolism. Veterinary applications such as mastitis management, wound healing, and antiviral prophylaxis may serve as intermediate translational steps, while Phase I/II clinical trials are ultimately required to verify therapeutic efficacy in humans.

In summary, this review integrates ethnomedicinal relevance, phytochemical diversity, and pharmacological potential of *C. nutans*, presenting a holistic perspective beyond earlier reports. By addressing methodological weaknesses and acknowledging toxicological gaps, it highlights future priorities including bioactivity-guided isolation, pharmacokinetic profiling, and veterinary applications. Ultimately, *C. nutans* emerges as a multi-constituent, multi-target candidate bridging traditional use with modern drug discovery.
